# Polyorchidism Presenting with Inguinal Hernia and Hypospadias

**DOI:** 10.1155/2013/917050

**Published:** 2013-08-07

**Authors:** Duygu Tatlı, Kemal Varım Numanoglu

**Affiliations:** Department of Pediatric Surgery, Faculty of Medicine, Bülent Ecevit University, Kozlu, 67600 Zonguldak, Turkey

## Abstract

Polyorchidism is defined as the presence of more than two testes. Triorchidism is the most frequent presentation. This anomaly is extremely rare, and approximately a hundred cases were described in the literature. We report a case of triorchidism presenting with inguinal hernia and penoscrotal hypospadias in a three-year-old male and briefly discuss current management of polyorchidism. Management remains controversial especially if there is no associated abnormality identified. The absence of any concomitant disorder and if testicular tumour can be ruled out by sonography or magnetic resonance imaging, surgical exploration with biopsy could be unnecessary. On the contrary, surgical exploration has the advantage of allowing for fixation of the testes to prevent torsion and determination of testicular outflow tracts and estimating reproductive capacity.

## 1. Introduction

Polyorchidism is defined as the presence of more than two testes, and it is a rare congenital abnormality. The first accepted case was a post mortem report by Ashfeld in 1880 [1] and since then approximately over a hundred cases have been reported in the literature. A majority of cases are triorchidism with occasional bilateral duplication [2]. We describe a case of triorchidism presenting with inguinal hernia and penoscrotal hypospadias in a three-year-old male and briefly discuss its management.

## 2. Case Presentation

**Figure 1:**
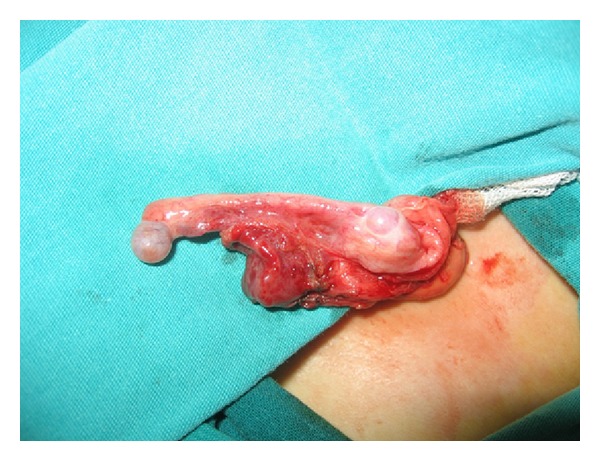
The small mass proximal to the left testicle proved to be a supernumerary testicle.

A three-year-old boy was admitted with the complaint of left inguinal swelling. On physical examination, a left sided inguinal hernia and penoscrotal hypospadias were found. Both testes were palpated in the scrotum. Other physical findings were normal. The family history was unremarkable. The diagnosis of right inguinal hernia and penoscrotal hypospadias was made. At the time of operation the right inguinal region was explored initially. Hernia sac and cord structures traversing the internal inguinal ring were identified. Exploration revealed two separate testicles within a single tunica vaginalis. Supernumerary testis was seen smaller than the other one, and it lacks an epididymis and vas (Figure 1). After performing high ligation and fixation of the normal testis into the scrotum, supernumerary testis was removed for a possible malignancy. Histopathological evaluation revealed presence of immature testicular tissue. The postoperative period was uneventful.

## 3. Discussion

Polyorchidism is an urogenital curiosity defined by the presence of more than two testes confirmed by histology. This anomaly is extremely rare, and approximately over a hundred cases were described in the literature. Although it can remain asymptomatic, polyorchidism is often associated to processus vaginalis anomalies and undescended testis in childhood. Supernumerary testes may have scrotal, inguinal, or abdominal location; they are more frequently on the left side, and their size is often smaller than both ipsilateral and contralateral testes [3]. As in the present case, triorchidism is the most frequent presentation. The cause of polyorchidism remains unclear. The etiology of polyorchidism is thought to be due to accidental longitudinal or transverse division of the genital ridge, with or without mesonephros, before the 8th week of gestational life, either through local accident or development of peritoneal bands. Depending on the segmentation plane and site, supernumerary testis may develop with a common or single epididymis and vas deferens. In most cases, the epididymis and vas deferens are shared or missing [4].

Thum [5] proposed a functional classification of polyorchidism based on embryonic development (Table 1). According these classifications, our patient was considered as Type 1.

Polyorchidism is usually identified during repair of inguinal hernia and orchidopexy in children. In our case, inguinal hernia and penoscrotal hypospadias were involved. Commonly associated anomalies are testicular maldescent (40%), inguinal hernia (30%), testicular torsion (13%), hydrocele (9%), and hypospadias (1%) [2]. Infertility is also a common finding (20%). There is an increased risk of malignancy if supernumerary testicles are detected. According to the literature, the risk of malignancy is estimated to be about 6%. The reported malignancy cases included seminoma, choriocarcinoma, and teratoma [6]. The polyorchid testis is among the differential possibilities under the category of extratesticular scrotal masses. Differential diagnosis includes spermatocele, hydrocele, epididymal cysts, fibrous pseudotumor, adenomatoid tumor, and papillary cystadenoma [7].

When a polyorchidism is suspected of palpable mass in the groin or scrotum, sonography is the effective, noninvasive modality for use in its investigation and preoperative evaluation. On sonography, an accessory testis usually displays a fine granular echotexture similar to that in the normal testis. Color doppler sonography can provide further information about the blood flow pattern in the testis. MRI may provide confirmation when the results of sonography are inconclusive [8].

The management of polyorchidism has been still controversial. In the past it was common practice to remove the supernumerary testicle with removal of the smaller mass [9]. More recently, with advances in ultrasound and magnetic resonance imaging technology, more conservative approaches have been recommended. In general, authors have either advocated surgical exploration or following with imaging modalities. Biopsy is a contentious issue and not routinely performed. A conservative approach relies on magnetic resonance imaging, and high resolution sonography is an effective, noninvasive means of accurately diagnosing polyorchidism. 

Some authors claim that conservative treatment is the appropriate choice. They suggest that supernumerary testis, even in ectopic locations, should be preserved if they appear normal and are potentially functional. They believed that the absence of any concomitant disorder and if testicular tumour can be ruled out by sonography or magnetic resonance imaging, surgical exploration with biopsy could be unnecessary [2, 5]. On the contrary, surgical exploration has the advantage of allowing for fixation of the testes to prevent torsion and determination of testicular outflow tracts and estimating reproductive capacity [10]. Indications for excision include malignant or dysplastic change on biopsy, ultrasound suggestive of malignancy, and absent reproductive potential of the polyorchid testis which lacks an epididymis or vas. In our case, the accessory testis showed no reproductive capacity due to a lack of attachment to a cord structure. It was therefore removed because of the high risk of malignancy.

## 4. Conclusion

The diagnosis of polyorchidism is usually incidental. According to our opinion, if polyorchidism is associated with doubtful concomitant pathology, surgical intervention should be undertaken. However, in uncomplicated polyorchidism, conservative management with close magnetic resonance imaging or ultrasonography observation should be recommended.

## Figures and Tables

**Table 1:** Functional classification of polyorchidism based on embryonic development (table derived from Thum [5]).

Type I	The supernumerary testis lacks an epididymis and vas. The split-off part of the primordial gonad does not communicate with the mesonephric tubules from which the epididymis develops.

Type II	The supernumerary testis is linked to the regular testis by a common epididymis and shares a common vas with it. The division of the genital ridge occurs in the region where the primordial gonads are attached to the mesonephric ducts, although the latter are not divided.

Type III	The supernumerary testis has its own epididymis but shares the vas with the regular testis.
